# Do pre-diagnosis primary care consultation patterns explain deprivation-specific differences in net survival among women with breast cancer? An examination of individually-linked data from the UK West Midlands cancer registry, national screening programme and Clinical Practice Research Datalink

**DOI:** 10.1186/s12885-017-3129-4

**Published:** 2017-02-23

**Authors:** M. Morris, L. M. Woods, K. Bhaskaran, B. Rachet

**Affiliations:** Faculty of Epidemiology and Population Health, Department of Non-Communicable Disease Epidemiology, London School of Hygiene & Tropical Medicine, Keppel St, London, WC1E 7HT UK

**Keywords:** Breast cancer, Socioeconomic inequalities, Primary care, Consultation, Survival, Early Diagnosis, Pathways, England

## Abstract

**Background:**

In England and Wales breast cancer survival is higher among more affluent women. Our aim was to investigate the potential of pre-diagnostic factors for explaining deprivation-related differences in survival.

**Methods:**

Individually-linked data from women aged 50–70 in the West Midlands region of England, diagnosed with breast cancer 1989–2006 and continuously eligible for screening, was retrieved from the cancer registry, screening service and Clinical Practice Research Datalink. Follow-up was to the end of July 2012. Deprivation was measured at small area level, based on the quintiles of the income domain of the English indices of deprivation. Consultation rates per woman per week, time from last breast-related GP consultation to diagnosis, and from diagnosis to first surgery were calculated. We estimated net survival using the non-parametric Pohar-Perme estimator.

**Results:**

The rate of primary care consultations was similar during the 18 months prior to diagnosis in each deprivation group for breast and non-breast symptoms. Survival was lower for more deprived women from 4 years after diagnosis. Lower net survival was associated with more advanced extent of disease and being non-screen-detected. There was a persistent trend of lower net survival for more deprived women, irrespective of the woman’s obesity, alcohol, smoking or comorbidity status. There was no significant variation in time from last breast symptom to diagnosis by deprivation. However, women in more deprived categories experienced significantly longer periods between cancer diagnosis and first surgery (mean = 21.5 vs. 28.4 days, *p* = 0.03). Those whose surgery occurred more than 12 weeks following their cancer diagnosis had substantially lower net survival.

**Conclusions:**

Our data suggest that although more deprived women with breast cancer display lifestyle factors associated with poorer outcomes, their consultation frequency, comorbidities and the breast cancer symptoms they present with are similar. We found weak evidence of extended times to surgical treatment among most deprived women who were not screen-detected but who presented with symptoms in primary care, which suggests that treatment delay may play a role. Further investigation of interrelationships between these variables within a larger dataset is warranted.

**Electronic supplementary material:**

The online version of this article (doi:10.1186/s12885-017-3129-4) contains supplementary material, which is available to authorized users.

## Background

In England and Wales there are substantial socio-economic differentials in breast cancer survival, with survival being relatively high among more affluent women [1, 2]. There is increasingly strong evidence that these are due in part to later stage of disease at presentation [3, 4] and differences in timeliness of diagnosis [5, 6]. Other potential drivers of late stage at diagnosis include health system delays in primary and secondary care (practitioner delays) [7, 8] and differences in patients’ underlying characteristics [9–11] and lifestyle choices [12–16].

Past research has focused on the appraisal by patients of the seriousness of their symptoms [10], and the possible sources of delay after presentation using patient experience surveys and audit data [17–20]. However, to date there has been little reporting of the connection between these patterns and the survival of the same women.

**Fig. 1:**
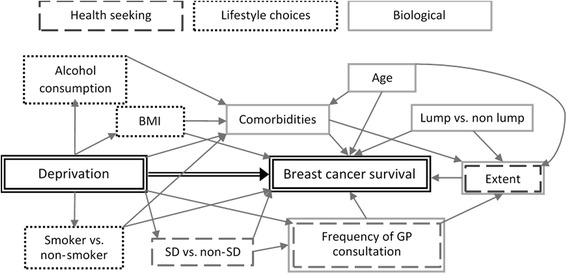
Conceptual framework for deprivation-specific differences in breast cancer survival. SD = screen-detected, non-SD = non-screen-detected

Our aim was to investigate the potential of pre-diagnostic factors for explaining deprivation differences in survival using the factors shown in the conceptual framework in Fig. 1. This shows the potential links between deprivation and the outcome of breast cancer survival, and patients’ biological, lifestyle, and health seeking characteristics. The primary outcomes examined were: the rate of primary care consultation; the time from last breast-related GP consultation to diagnosis, and from diagnosis to first surgery; and net survival. We first describe deprivation-specific variations in women’s baseline characteristics, pre-diagnostic primary care consultations patterns, symptom presentation, and time intervals from symptom report to diagnosis. We then examine how net survival varies with deprivation and with each of these covariates. To do so, we used individually-linked data from primary care, the screening service and the cancer registry.

## Methods

### Data sources and study population

The West Midlands Breast Screening Quality Assurance Reference Centre provided data on a cohort of women aged 50–70, diagnosed with primary malignant breast cancer from 1 April 1989 to 31 March 2006 and eligible for screening. This dataset included women’s screening status, their deprivation category and inpatient hospital stays from Hospital Episode Statistics (HES). Follow-up was complete up to the end of July 2012 for all women. A subset of these data was linked to the General Practice Research Database (GPRD, now subsumed into the Clinical Practice Research Datalink, CPRD), which contains routinely collected data from primary care including symptoms, diagnoses, tests, therapies and lifestyle-related information. Only women registered in a practice which had met a set of basic data quality criteria for the full 12 months prior to the woman’s diagnosis were included.

### Variables

#### Deprivation

Deprivation was measured using the income domain of the English indices of deprivation derived from administrative data pertaining to the years 2001, 2005 and 2008 [21–23]. These scores are defined for the Lower Super Output Areas that existed at the 2001 census (LSOAs, approximately 1500 people). Each woman was assigned to a deprivation category derived from the score temporally closest to her date of diagnosis and on the basis of her address of residence. The scores were split in five categories based on the quintiles of the national distribution of the areas. Women were grouped for some analyses into two categories: less deprived (quintiles 1 and 2) and middle and more deprived (quintiles 3, 4 and 5).

#### Other covariables

Age at diagnosis was grouped into five-year age bands. Women were categorised as either “screen-detected” or “not screen-detected”. The latter included women diagnosed with interval cancers (when a women’s last screening attendance had resulted in a negative screen and she had not yet been invited to a subsequent screening), lapsed and non-attenders at screening. These are the women whose cancer was detected symptomatically.

Using information on tumour size, node involvement and presence of metastases from the cancer registry database, we defined “extent of disease” for each woman as: *localised* (confined to the organ of origin), *regional* (spread to adjacent muscle, organ, fat, connective tissue or regional lymph nodes), and *distant* (distant metastases).

Patient characteristics for each woman were derived from the CPRD data. Smoking (non- or ex-smoker, current smoker), alcohol consumption status (non-, ex-, current drinker), and body mass index (BMI; <25 kg/m^2^, 25 to <30 kg/m^2^, 30+ kg/m^2^ [24, 25]) were all extracted from the patient records. Comorbidities, identified from the CPRD records using lists of National Health Service Read Codes (which are the principal method of coding clinical information in primary care), were combined into a Charlson score [26]. The Charlson score was also derived from the HES data. The higher of the two (HES vs. CPRD) was taken in each case as the patients’ final comorbidity score.

#### Primary care consultations

Primary care consultations in the 18 months before diagnosis were derived from the CPRD data. These were categorised as “breast-related” or “not breast-related”. A breast-related consultation was one which included any mention of a breast symptom in the Read code list [27]. We also included those where the doctor had noted “mammography”, “female cancer of the breast”, “breast examination” and other breast-related codes. These were identified by searches of Read code descriptions (Additional file 1). These were not all necessarily related to symptom presentation, but some would have indicated a suspected diagnosis. However, they all pre-dated histologically-confirmed diagnosis and so were counted as a consultation about the breast that might have then led to diagnosis of breast cancer. Breast symptoms were grouped into those involving a lump (including fibroadenoma, nodularity, cyst, axillary and cervical lymphadenopathy), or not (nipple-related: bleeding, retraction, discharge, Pagets’ nipple, pain; and other: skin changes, breast infection, breast pain). Time in days from a woman’s last breast-related consultation at the GP to diagnosis, and from diagnosis to first surgery (within 18 months) were calculated for women whose cancer was not detected through screening and who also reported a breast symptom in primary care.

### Statistical analysis

Consultation rates per woman per week for both breast and non-breast symptoms were calculated along with their 95% confidence intervals. Chi-squared tests and non-parametric tests for trend for continuous variables [28] were used to examine the association between deprivation and co-variables. Small numbers precluded multivariable analyses for these data. All analyses were carried out in Stata 14 [29].

We estimated net survival for women using the non-parametric Pohar-Perme estimator [29, 30] by deprivation and for each covariable. We stratified the net survival estimates for each deprivation group by each covariable. Net survival provides an estimate of survival from the cancer itself, adjusting for expected mortality from other causes. We derived individual estimates of expected mortality from ethnic-specific deprivation-adjusted life tables for England and Wales [31]. We applied locally-weighted regression to smooth the survival estimates [32, 33] with a conservative degree of smoothing to maintain the variability evident in the more sparse data. Where data were very sparse, smoothing was not performed.

## Results

### Sample

Among 28,885 women in the West Midlands dataset, 786 (2.72%) could be linked to CPRD primary care record. The matching proportion ranged from 0% in 1989 to a maximum of 4.96% in 2002, and was 3.25% in the last year of data available, 2006. The matched sample of women were similar to the cohort in relation to their age and distribution of extent of disease at diagnosis. Fewer women were diagnosed in the period before 1995 (*p* < 0.001) and a greater proportion were screen-detected, both reflecting the increasing coverage of the CPRD over time (Additional file 1: Table S1A). Women in the sample were more likely to be alive at the end of follow-up (*p* < 0.001), and less likely to be deprived (*p* < 0.001). Net survival was comparable overall and by deprivation (Additional file 1: Figure S1A).

Sixty women had no recorded consultations in surgery with a doctor in the 18 months before diagnosis. Of the remaining 726 patients, the number of consultations with a doctor ranged from 1 to 52, with a mean of 14.3. Half of all women reported no breast symptoms in the primary care setting during the 18 months before diagnosis (50.1%), whilst 40.8% had one breast-related consultation. The remaining 9.0% had more than one breast-related consultation, with a mean of 2.4 per woman and a maximum of 6.

### Consultation rate

**Fig. 2:**
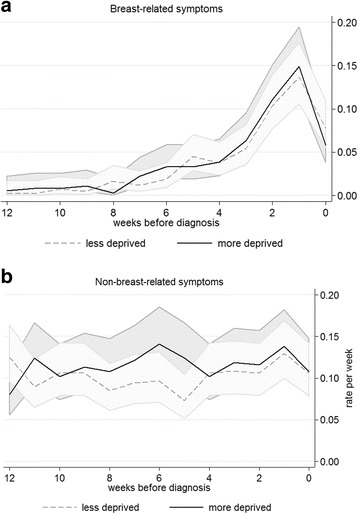
Consultation rates in the 12 weeks prior to diagnosis by deprivation: women diagnosed in West Midlands with invasive breast cancer 1989–2006 found within the CPRD dataset (*N* = 786); **a** breast-related symptoms, **b** non-breast-related symptoms

The rate of consultation was similar during the 18 months prior to diagnosis in each deprivation group, for both breast and non-breast symptoms (Fig. 2). A rise in the overall rate of consultations was seen from 3 months before diagnosis for breast-related consultations. The majority of the rise in breast-related consultations was among women whose cancer was not screen-detected. Consultation rates did not vary by age, period, extent of disease, BMI, smoking or alcohol consumption.

### Associations between co-variables and deprivation

Women in the more deprived groups were more likely to have had their cancer identified through screening (*p* = 0.013), and were more likely to have regional or distant spread of disease (*p* = 0.045). They were also less likely to be alive at the end of follow up (*p* = 0.008) (Table 1). In addition, the primary care data showed that deprived women were more likely to be current or ex-smokers, have a higher BMI, but were less likely to be current drinkers. There was no association between deprivation and having a comorbidity at diagnosis, nor the number of distinct breast symptoms reported, nor whether those who had a breast-related consultation presented with a lump symptom or not (Table 2).

**Table 1:** Distribution of co-variables by deprivation quintile among all women (*N* = 786)

	Total	Least deprived	2	3	4	Most deprived	*p*-value^a^
	n (%)	n (%)	n (%)	n (%)	n (%)	n (%)	
Total	786 (100)	211 (100)	213 (100)	140 (100)	112 (100)	110 (100)	
Vital status at end of follow up							
Alive	567 (72.1)	165 (78.2)	154 (72.3)	106 (75.7)	76 (67.9)	66 (60.0)	0.008
Dead	219 (27.9)	46 (21.8)	59 (27.7)	34 (24.3)	36 (32.1)	44 (40.0)	
Age groups							
50–54	191 (24.3)	49 (23.2)	57 (26.8)	29 (20.7)	27 (24.1)	29 (26.4)	0.707
55–59	174 (22.1)	48 (22.7)	48 (22.5)	35 (25.0)	21 (18.8)	22 (20.0)	
60–64	216 (27.5)	66 (31.3)	58 (27.2)	33 (23.6)	33 (29.5)	26 (23.6)	
65–70	205 (26.1)	48 (22.7)	50 (23.5)	43 (30.7)	31 (27.7)	33 (30.0)	
Extent of disease at diagnosis							
Localised	437 (55.6)	127 (60.2)	118 (55.4)	81 (57.9)	59 (52.7)	52 (47.3)	0.045
Regional	255 (32.4)	64 (30.3)	65 (30.5)	45 (32.1)	39 (34.8)	42 (38.2)	
Distant	24 (3.1)	3 (1.4)	5 (2.3)	3 (2.1)	4 (3.6)	9 (8.2)	
Missing	70 (8.9)	17 (8.1)	25 (11.7)	11 (7.9)	10 (8.9)	7 (6.4)	
Screening groups							
Screen-detected cancer	316 (40.2)	112 (53.1)	119 (55.9)	64 (45.7)	65 (58.0)	74 (67.3)	0.013
Not screen-detected cancer	434 (55.2)	91 (43.1)	83 (39.0)	67 (47.9)	45 (40.2)	30 (27.3)	
Missing	36 (4.6)	8 (3.8)	11 (5.2)	9 (6.4)	2 (1.8)	6 (5.5)	
Period of diagnosis							
1989–1994	81 (10.3)	18 (8.5)	22 (10.3)	13 (9.3)	11 (9.8)	17 (15.5)	0.011
1995–2000	283 (36.0)	79 (37.4)	64 (30.0)	57 (40.7)	32 (28.6)	51 (46.4)	
2001–2006	422 (53.7)	114 (54.0)	127 (59.6)	70 (50.0)	69 (61.6)	42 (38.2)	
Smoking status							
Non- or ex-smoker	541 (68.8)	159 (75.4)	154 (72.3)	94 (67.1)	74 (66.1)	60 (54.5)	0.002
Current smoker	222 (28.2)	45 (21.3)	55 (25.8)	42 (30.0)	33 (29.5)	47 (42.7)	
Missing	23 (2.9)	7 (3.3)	4 (1.9)	4 (2.9)	5 (4.5)	3 (2.7)	
Alcohol consumption status							
Non-drinker	84 (10.7)	17 (8.1)	13 (6.1)	19 (13.6)	12 (10.7)	23 (20.9)	<0.001
Current drinker	569 (72.4)	161 (76.3)	171 (80.3)	96 (68.6)	72 (64.3)	69 (62.7)	
Ex-drinker	81 (10.3)	19 (9.0)	16 (7.5)	14 (10.0)	18 (16.1)	14 (12.7)	
Missing	52 (6.6)	14 (6.6)	13 (6.1)	11 (7.9)	10 (8.9)	4 (3.6)	
BMI (kg/m^2^)							
< 25 kg/m^2^	305 (38.8)	84 (39.8)	99 (46.5)	53 (37.9)	40 (35.7)	29 (26.4)	0.001
25 to <30	235 (29.9)	73 (34.6)	50 (23.5)	43 (30.7)	32 (28.6)	37 (33.6)	
30+	189 (24)	41 (19.4)	49 (23.0)	29 (20.7)	32 (28.6)	38 (34.5)	
Missing	57 (7.3)	13 (6.2)	15 (7.0)	15 (10.7)	8 (7.1)	6 (5.5)	
Charlson score							
0	684 (87)	191 (90.5)	185 (86.9)	124 (88.6)	93 (83.0)	91 (82.7)	0.202
1+	102 (13)	20 (9.5)	28 (13.1)	16 (11.4)	19 (17.0)	19 (17.3)	

^a^ *P*-values are derived from *X* ^2^ tests for categorical variables and non-parametric tests for trend for continuous variables (BMI, breast-related consultations). All tests exclude missing values

**Table 2:** Distribution of co-variables by deprivation quintile among women who were not screen-detected (*N* = 434)

	Total	Least deprived	2	3	4	Most deprived	*p*-value^a^
	n (%)	n (%)	n (%)	n (%)	n (%)	n (%)	
Total	434 (100)	112(100)	119 (100)	64 (100)	65 (100)	74 (100)	
Number of distinct breast symptoms reported in 18mths before diagnosis							
0	103 (23.7)	37 (33.0)	22 (18.5)	12 (18.8)	16 (24.6)	16 (21.6)	0.137
1	278 (64.1)	66 (58.9)	77 (64.7)	45 (70.3)	39 (60.0)	51 (68.9)	
2+	53 (12.2)	9 (8.0)	20 (16.8)	7 (10.9)	10 (15.4)	7 (9.5)	
Lump symptom							
No	137 (31.6)	43 (38.4)	38 (31.9)	11 (17.2)	22 (33.9)	23 (31.1)	0.069
Yes	297 (68.4)	69 (61.6)	81 (68.1)	53 (82.8)	43 (66.2)	51 (68.9)	

^a^ *P*-values are derived from *X* ^2^ tests for categorical variables and non-parametric tests for trend for continuous variables (BMI, breast-related consultations). All tests exclude missing values

### Net survival by deprivation

**Fig. 3:**
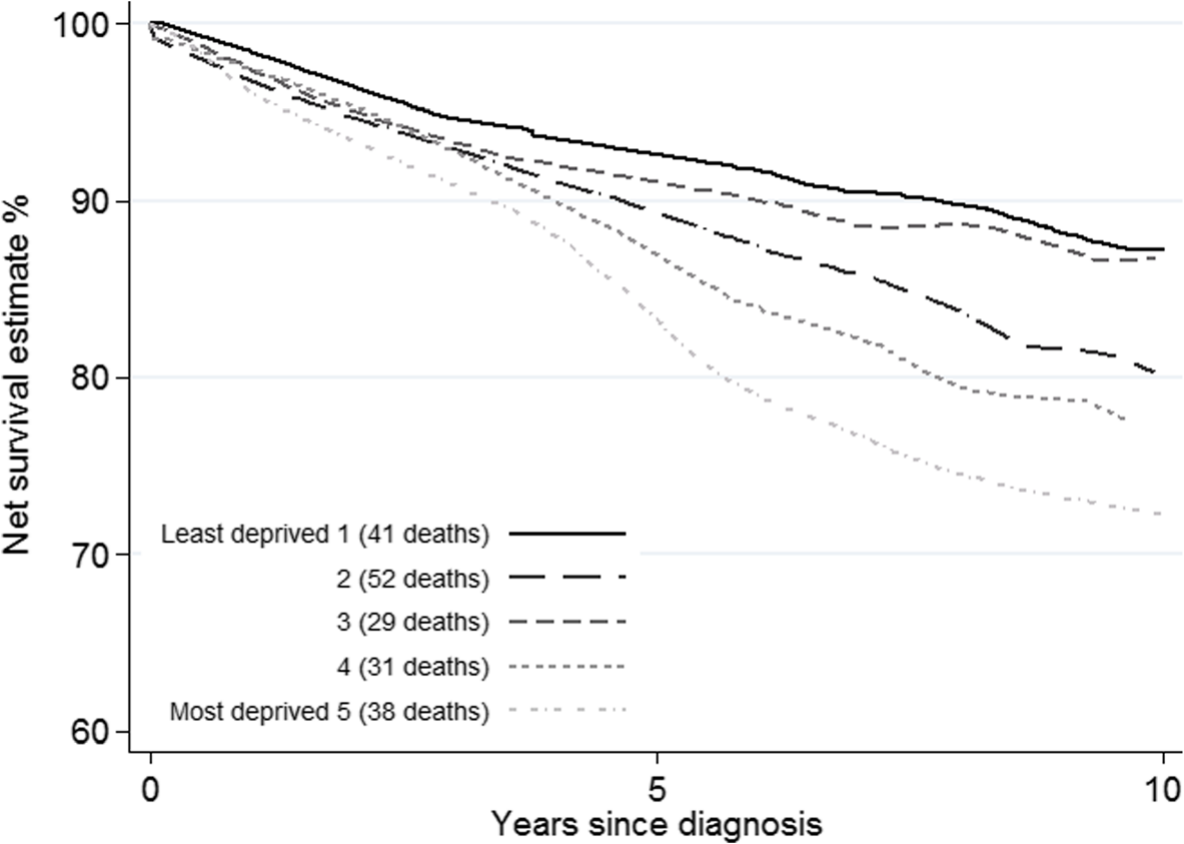
Net survival by deprivation: all women (*N* = 786). Footnotes: 95% CIs overlap, so are not displayed for clarity. Survival from date of cancer diagnosis to death, or the end of follow-up

Survival was lower for more deprived women from 4 years after diagnosis (Fig. 3). There was a suggestion that more deprived groups experienced worse survival, though the apparent trend was compatible with chance variation.

### Net survival by covariables

Lower net survival was associated with more advanced extent of disease and not being screen-detected. There was some evidence that reporting a non-lump symptom led to poorer survival (among those not screen-detected who reported a symptom).

**Fig. 4:**
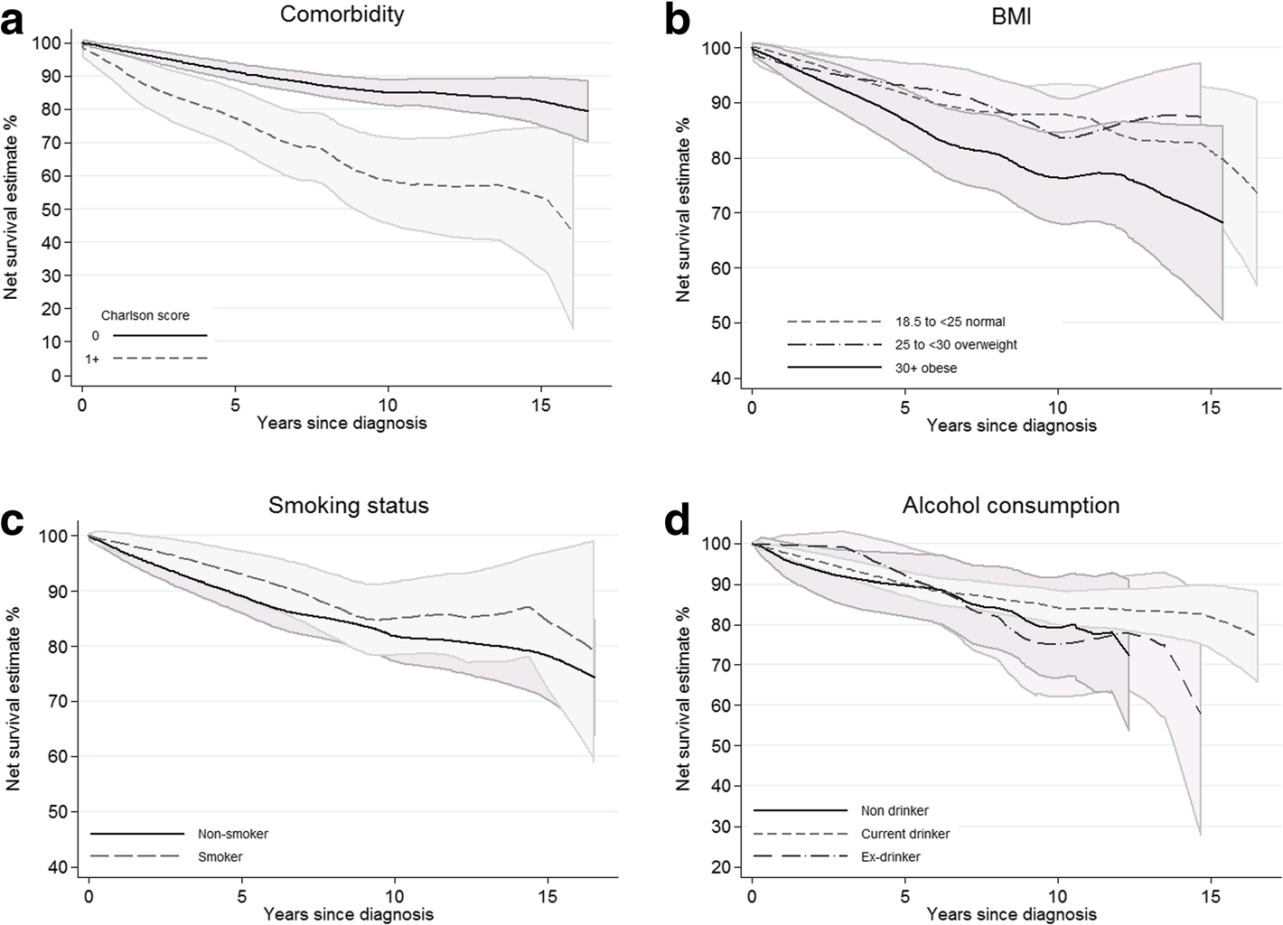
Net survival for all women in the sample (*N* = 786) by (**a**) the presence of comorbidities, (**b**) BMI, (**c**) smoking status and (**d**) alcohol consumption. Footnotes: Estimates smoothed. Survival from date of cancer diagnosis to death, or the end of follow-up

The presence of comorbidity was strongly associated with poorer net survival, especially over longer time periods (Fig. 4a), and there was also some evidence that obesity was also associated with poorer outcomes (Fig. 4b). There was little evidence of difference in net survival from breast cancer by smoking status, or by alcohol consumption status (Fig. 4c and d).

We examined the potential interaction between deprivation and each of these co-variables by deriving net survival for each of their sub-groups. Throughout, there was a persistent trend of lower net survival for more deprived women. This disadvantage was present irrespective of the woman’s obesity, alcohol, smoking or comorbidity status (Additional file 1: Figure S2B). Furthermore, the magnitude of the survival difference was similar for all co-variables.

### Time from last breast-related consultation to diagnosis and from diagnosis to surgery

We examined variations in the time elapsed from last symptom report (last breast-related consultation) to cancer diagnosis among women whose cancer was not screen-detected *and* who reported breast symptoms in primary care (*N* = 331). We also analysed the time elapsed from diagnosis to first surgery within 18 months of diagnosis for women whose diagnosis date was recorded earlier than the date of their surgery (*N* = 212).

There was no significant variation in time from last breast symptom to diagnosis by deprivation or any other co-variable (Table 3), although women in the most deprived group appeared to have longer waiting times than the other four quintiles, as did obese women, and women who did not present with a breast lump.

**Table 3:** Mean time from last breast symptom reported to diagnosis (*N* = 331) and to surgery (*N* = 212) by co-variables, among non-screen-detected women who also reported breast symptoms

	Time from last breast-related consultation to diagnosis			Time from diagnosis to surgery		
	N (%)	Mean (range), days	*P* value (trend)	N (%)	Mean (range), days	*P* value (trend)^a^
Total	331 (100.0)	16.0 (1–509)		212 (100.0)	23.7 (1–199)	
Deprivation						
Least deprived	75 (22.7)	15.6 (1–444)	0.299	48 (22.6)	22.1 (7–117)	0.034
2	97 (29.3)	15.1 (1–509)		59 (27.8)	21.5 (3–156)	
3	52 (15.7)	15.8 (2–116)		34 (16.0)	20.9 (1–162)	
4	49 (14.8)	15.0 (2–373)		35 (16.5)	27.6 (13–136)	
Most deprived	58 (17.5)	19.3 (1–389)		36 (17.0)	29.2 (10–199)	
Extent						
1	162 (48.9)	14.6 (1–444)	0.554	110 (51.9)	22.0 (3–139)	0.137
2	134 (40.5)	17.2 (1–509)		89 (42.0)	24.3 (1–199)	
3	12 (3.6)	13.9 (2–222)		4 (1.9)	48.5 (22–156)	
missing	23 (6.9)			9 (4.2)		
Age						
50–54	89 (26.9)	19.4 (1–509)	0.144	59 (27.8)	21.8 (2–162)	0.790
55–59	62 (18.7)	14.6 (1–258)		45 (21.2)	25.2 (3–153)	
60–64	89 (26.9)	15.9 (2–109)		54 (25.5)	23.9 (1–151)	
65–70	91 (27.5)	14.1 (1–389)		54 (25.5)	24.3 (6–199)	
BMI						
< 25 normal	141 (42.6)	15.0 (1–444)	0.235	85 (40.1)	21.3 (2–199)	0.590
25to < 30 overweight	97 (29.3)	14.5 (1–509)		68 (32.1)	26.7 (7–156)	
30+ obese	68 (20.5)	20.1 (4–444)		49 (23.1)	22.4 (1–162)	
missing	25 (7.6)			10 (4.7)		
Smoking						
non- or ex-smoker	219 (66.2)	15.4 (1–509)	0.371	142 (67.0)	23.4 (1–156)	0.881
current smoker	102 (30.8)	17.5 (1–258)		64 (30.2)	24.3 (3–199)	
missing	10 (3.0)			6 (2.8)		
Alcohol consumption status						
non-drinker	37 (11.2)	14.0 (2–101)	0.989	26 (12.3)	21.6 (11–53)	0.857
current drinker	242 (73.1)	16.4 (1–509)		155 (73.1)	24.1 (1–199)	
ex-drinker	33 (10.0)	15.4 (2–393)		23 (10.8)	21.3 (6–106)	
missing	19 (5.7)			8 (3.8)		
Charlson score						
0	298 (90.0)	15.8 (1–509)	0.414	188 (88.7)	23.1 (1–199)	0.103
1+	33 (10.0)	17.4 (2–109)		24 (11.3)	28.9 (12–106)	
Lump symptom						
No	59 (17.8)	20.0 (1–444)	0.206	35 (16.5)	23.1 (3–199)	0.827
Yes	272 (82.2)	15.2 (1–509)		177 (83.5)	23.8 (1–162)	

^a^ Non-parametric tests for trend statistics and means exclude missing values and were performed on log-transformed values

By contrast, there was evidence that women in more deprived categories experienced longer periods between cancer diagnosis and first surgery: 21.5 days in quintiles 1, 2 and 3 compared to 28.4 days for quintiles 4 and 5 (*p*-value 0.03). Among women in the study, those diagnosed with distant disease had longer mean time to surgery compared with those with either regional or localised disease, but this difference may have reflected chance variation (*p* = 0.14).

**Fig. 5:**
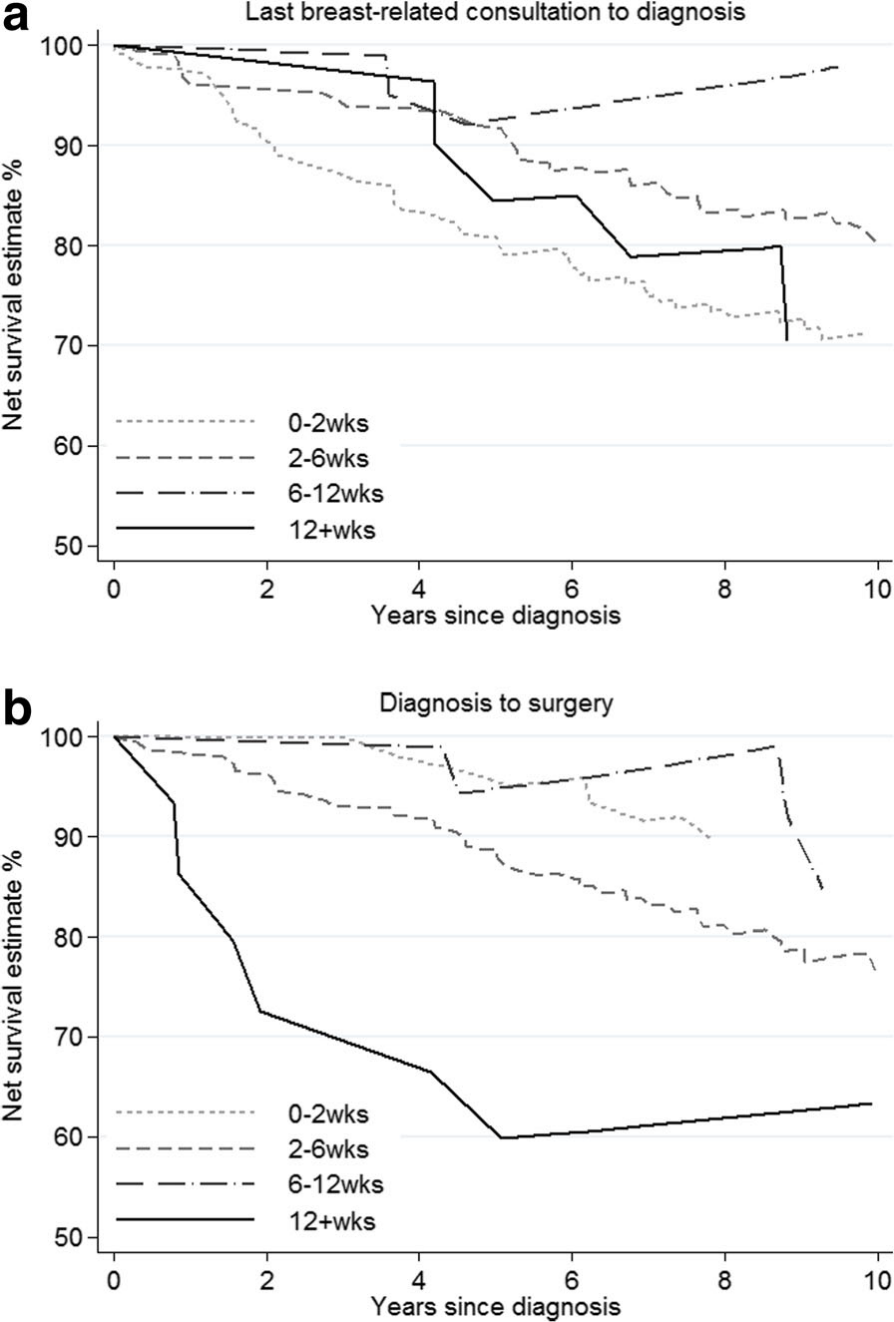
Net survival by time from last symptom to (**a**) diagnosis (*N* = 331) and (**b**) surgery (*N* = 212) among those symptomatically detected and who reported breast symptoms prior to diagnosis. Footnotes: Estimates unsmoothed due to sparsity of some data. CIs not shown for clarity. Some survival curves rise over time because in certain intervals the survival of the cancer patients is better than the population from which they are drawn, thus the interval-specific estimate of net survival is greater than 100%. Survival from date of cancer diagnosis to death, or the end of follow-up

Among these same non-screen-detected women there was little evidence of survival differences by time from last breast-related consultation to diagnosis within the first 5 years. After this, women for whom the time interval from consultation to diagnosis was 43–84 days (6–12 weeks) had higher survival, although not significantly so (Fig. 5a). Conversely, those whose surgery occurred more than 12 weeks following their cancer diagnosis had substantially lower net survival compared to those whose surgery took place within 12 weeks (Fig. 5b). This difference was evident from cancer diagnosis onwards.

## Discussion

Our data suggest that although more deprived women display lifestyle factors associated with poorer outcomes, their consultation frequency for both breast and non-breast symptoms, their comorbidities and the type of breast cancer symptoms they present with are similar. This interpretation is supported by the lack of difference in consultation patterns by extent of disease, BMI, smoking or alcohol consumption status coupled with the persistence of deprivation differences in survival within the sub-groups of these co-variables. We found some evidence of extended times to surgical treatment among most deprived women who were not screen-detected but who presented with symptoms in primary care. This potentially suggests that treatment delay may play a role, either due to a greater proportion of deprived women receiving neo-adjuvant treatment, or due to differences in the way more deprived patients interact with the healthcare system. Further investigation of the interrelationships between these variables within a larger and more detailed dataset is warranted.

These data were from a centre of excellence in breast cancer registration, and although small numbers limited our ability to perform multivariable analyses, our sample was representative, in terms of survival and most other characteristics, of the cohort from which they were drawn. The sample was somewhat less deprived than the cohort of women from which they came which is likely to be due to the profiles of the GP practices that contributed to the CPRD dataset within the West Midlands. In most cases there was less than 10% missing data in the analysis. The data available included women diagnosed some time ago, however, despite the changes in the healthcare system in the years since these data were collected, this study highlights some important areas that are less likely to have changed greatly. Despite a persistent deprivation gap in survival, we have found no difference in rate of consultation between the more and less deprived. In addition, changes in the system have resulted in almost unchanged high proportions of diagnoses after emergency admission for certain cancers (such as colon and lung).

Women who were not screen-detected and who presented with symptoms in primary care are those for whom policies encouraging early diagnosis are likely to be most effective. However, time from last breast-related consultation to diagnosis in this group was not significantly related to any of the variables investigated. This may suggest that patient delay prior to diagnosis is not such an important driver of survival differences within this cohort as might be assumed. On the other hand, time from last consultation to surgery in this group was longer among more deprived women, overweight or obese women, those who had a comorbidity, and those who were older. This potentially suggests that symptomatically-detected women are being referred and diagnosed just as quickly, regardless of their socio-demographic characteristics, but that differences in time to surgical treatment emerge after diagnosis. Such differences might be in part due to greater complexity in preparing more comorbid women, or women with more advanced stage of disease, for surgery, or due to differences in the way more deprived women navigate the healthcare system. In order to be able to draw firm conclusions about this, we would need specific information on treatments received including the rationale behind decisions made, in particular in relation to timing, adjuvant therapies administered, patient input into these decisions, and patient adherence. Reliable recurrence information for each woman over a medium to long period of follow-up would also be required.

There are some well-established relationships between deprivation and smoking, BMI, alcohol consumption, screening and stage at diagnosis [12–16, 34–36] but few have been investigated using the primary care data to date. BMI was associated in this sample with both deprivation and time to surgery, and may therefore explain some of the relationship between deprivation and survival. Conversely, the presence of comorbidities, while closely associated with survival and time to surgery, was not significantly related to deprivation. This contrasts with previous work where comorbidity was more frequent among the more deprived [37, 38]. However, in our data very few women had more than one comorbidity, probably because we included only women aged 50–70 (those eligible for breast screening). These women are generally healthier than the older women with breast cancer.

## Conclusions

We have previously shown that differences in survival by deprivation are evident among both women whose cancer was screen-detected and those not screen-detected [36] and that these differences are not entirely explained by adjustment for stage of disease and treatment received [39]. In those analyses we made corrections for potential biases due to lead time [40] and over-diagnosis.

This preliminary exploration of a linked sub-sample of these same data has highlighted both similarities and differences in the characteristics of women by deprivation status. It has shown a reassuring lack of difference in the impact of deprivation on consultation patterns, and a lack of association between lifestyle factors associated with deprivation and survival. On the other hand, our data point to some potential differences in time to surgical treatment that should be explored further. Our analysis demonstrates the feasibility of using primary care data to add to our understanding of what underlies survival differences. Larger samples linked to richer data on treatment decisions and pathways are required to elucidate these patterns further.

## Additional file

Additional file 1: Supplementary materials. (PDF 1260 kb)

## Abbreviations

BMI: Body mass index
CPRD: Clinical Practice Research Datalink
GPRD: General Practice Research Database
HES: Hospital Episode Statistics
LSOA: Lower Super Output Area
