# Supplementary material for: Do pre-diagnosis primary care consultation patterns explain deprivation-specific differences in net survival among women with breast cancer? An examination of individually-linked data from the UK West Midlands cancer registry, national screening programme and Clinical Practice Research Datalink
Source: BMC Cancer. 2017 Feb 23;17:155. doi: 10.1186/s12885-017-3129-4 (PMC5324281; doi:10.1186/s12885-017-3129-4)
Supplement: Supplementary file 1 — Supplementary materials. (PDF 1260 kb) [file 12885_2017_3129_MOESM1_ESM.pdf]

Additional file 1

Breast symptoms included in "breast-related consultations"

"Symptom library" for breast-related symptoms supplied by Prof W Hamilton's Primary Care Diagnostics Group (Exeter University), which consists of codes recorded by the GP during consultations that have been identified as related to breast symptoms (Walker *et al*, 2014).

- Breast lump
- Breast fibroadenoma
- Breast pain
- Breast nodularity
- Nipple bleed
- Nipple retraction
- Nipple discharge
- Paget's nipple
- Nipple pain
- Breast skin changes
- Breast cyst
- Breast infection
- Breast other
- Axilliary lymphadenopathy
- Cervical lymphadenopathy

Other breast-related codes found in text descriptions of other codes

(NOS= not otherwise specified; NAD = no appreciable disease)

- Malignant neoplasm of female breast
- Ca female breast
- Breast examination
- Breast signs and symptoms NOS
- Mammography - X-ray
- Mammography abnormal
- Axillary pain
- Breast signs and symptoms
- Disorders of breast
- Eversion of nipple
- Family History: Breast cancer
- Inflammatory breast diseases NOS
- General breast exam. NAD
- General breast exam. NOS
- Other breast injuries
- Referral to mammography clinic
- Suspected breast cancer
- Axillary lump

Table S1A: Sample characteristics (N=786) compared to the remaining cohort (N=28,885)

		Sample matched to CPRD		Remaining cohort		
		n	%	N	%	χ^{2} [a] p value
**Total**		**786**	**100.0**	**28,885**	**100**	
**Mean age (SD)**			59.9 (6.0)		59.7 (6.0)	
**Age groups**						
	50-54	191	24.3	7,169	24.8	0.310
	55-59	174	22.1	7,079	24.5	
	60-64	216	27.5	7,264	25.1	
	65-70	205	26.1	7,373	25.5	
**Vital status**						
	Alive	567	72.1	17,202	59.6	<0.001
	Dead	219	27.9	11,683	40.4	
**Extent of disease at diagnosis**						
	Localised	437	55.6	15,785	54.6	0.940
	Regional	255	32.4	9,108	31.5	
	Distant	24	3.1	924	3.2	
	Missing	*70*	*8.9*	*3,068*	*10.6*	
**Screening groups**						
	Screen-detected cancer	316	40.2	10,584	36.6	0.041
	Not screen-detected cancer	470	59.8	18,301	63.4	
**Deprivation category**						
	Least deprived	211	26.8	5,307	18.4	<0.001
	2	213	27.1	6,470	22.4	
	3	140	17.8	5,750	19.9	
	4	112	14.2	5,414	18.7	
	Most deprived	110	14.0	5,916	20.5	
	Missing	*0*	*0*	*28*	*0.1*	
**Period of diagnosis**						
	1989-1994	81	10.3	8,173	28.3	<0.001
	1995-2000	283	36.0	9,428	32.6	
	2001-2006	422	53.7	11,284	39.1	

[a] χ^{2} test performed on non-missing data only

Figure S1A: Comparison of net survival of matched sample to target population cohort overall and by deprivation

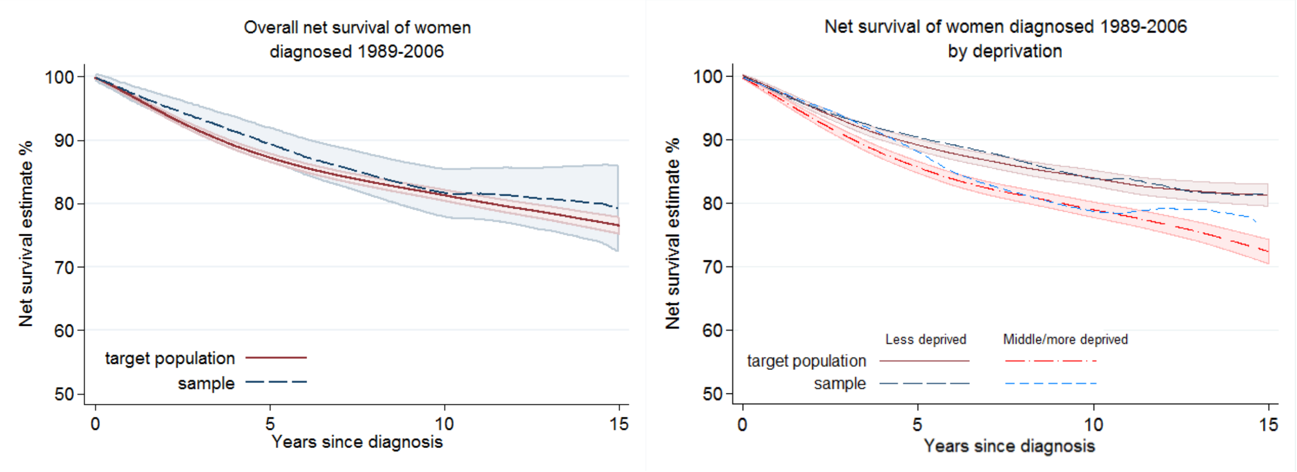

Footnote: Estimates smoothed. CIs shown only for target population for clarity. Sample CIs fully overlap with those of target population.

Figure S2B: Net survival by deprivation (quintiles 1+2 vs quintiles 3,4+5) and co-variable sub-group

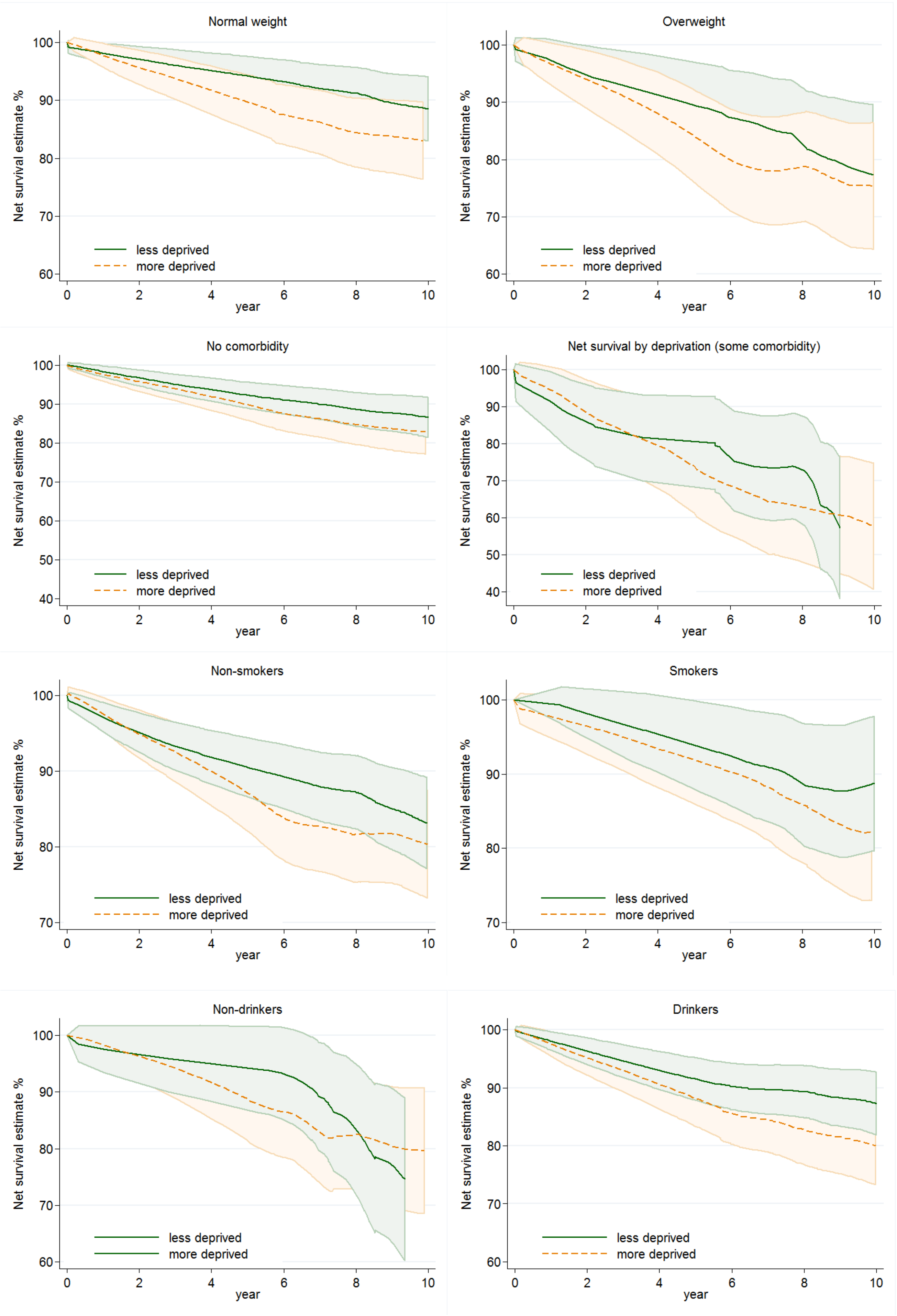

Footnote: Net survival estimated for all women with non-missing data, by co-variable subgroups shown in Table 1. Estimates smoothed.
